# Supplementary material for: Contextualising COVID-19 prevention behaviour over time in Australia: Patterns and long-term predictors from April to July 2020 in an online social media sample
Source: PLoS One. 2021 Jun 29;16(6):e0253930. doi: 10.1371/journal.pone.0253930 (PMC8241082; doi:10.1371/journal.pone.0253930)
Supplement: S1 Table — (DOCX) [file pone.0253930.s001.docx]

**S1 Table. Descriptive characteristics of analysis sample (N=1843) and full sample invited for follow-up (N=3216) at baseline (April)****.**

| **Characteristic** | | **Analysis sample (N=1843) n (%)** | **Full sample (N=3216) n (%)** |
| --- | --- | --- | --- |
| Age group | |  |  |
|  | 18 to 25 years | 353 (19.2%) | 800 (24.9%) |
|  | 26 to 40 years | 528 (28.6%) | 930 (28.6%) |
|  | 41 to 55 years | 462 (25.1%) | 737 (22.9%) |
|  | 56 to 90 years | 500 (27.1%) | 749 (23.3%) |
| Gender | |  |  |
|  | Male | 487 (26.4%) | 1120 (35.0%) |
|  | Female | 1322 (71.7%) | 2033 (63.5%) |
|  | Other/prefer not to say | 34 (1.8%) | 48 (1.5%) |
| Educational attainment* | |  |  |
|  | Less than university | 496 (26.9%) | 971 (30.3%) |
|  | University | 1347 (73.1%) | 2230 (69.7%) |
| State/territory of residence | |  |  |
|  | Australian Capital Territory | 58 (3.1%) | 95 (3.0%) |
|  | Northern Territory | 7 (0.4%) | 16 (0.5%) |
|  | Victoria | 291 (15.8%) | 495 (15.5%) |
|  | New South Wales | 937 (50.8%) | 1664 (52.0%) |
|  | Queensland | 258 (14.0%) | 447 (14.0%) |
|  | Western Australia | 130 (7.1%) | 221 (6.9%) |
|  | South Australia | 84 (4.6%) | 143 (4.5%) |
|  | Tasmania | 78 (4.2%) | 120 (3.7%) |
| Residential area remoteness^ | |  |  |
|  | Major cities | 1374 (74.6%) | 2399 (75.5%) |
|  | Regional and remote | 467 (25.4%) | 780 (24.5%) |
| Socioeconomic status, mean IRSAD quintile (SD) | | 3.66 (1.40) | 3.67 (1.39) |
| Born in Australia | | 1405 (76.2%) | 2425 (75.8%) |
| English primary language | | 1774 (96.3%) | 3025 (94.5%) |
| Aboriginal or Torres Strait Islander | |  |  |
|  | Yes | 31 (1.7%) | 57 (1.8%) |
|  | No | 1796 (97.4%) | 3109 (97.1%) |
|  | Did not respond | 16 (0.9%) | 35 (1.1%) |
| Chronic health conditions | |  |  |
|  | None | 912 (49.5%) | 1683 (52.3%) |
|  | One | 537 (29.1%) | 910 (28.3%) |
|  | Two or more | 394 (21.4%) | 623 (19.4%) |
| Health literacy adequacy^†^ | | 1695 (92.0%) | 2880 (90.0%) |
| Self-Reported General Health | |  |  |
|  | Poor | 68 (3.7%) | 102 (3.2%) |
|  | Fair | 248 (13.5%) | 442 (13.8%) |
|  | Good | 629 (34.1%) | 1099 (34.3%) |
|  | Very good | 667 (36.2%) | 1144 (35.7%) |
|  | Excellent | 231 (12.5%) | 414 (12.9%) |

*Notes:* The analysis sample comprised participants from our prospective longitudinal study who provided responses to the baseline survey (April) and at least one subsequent follow-up survey (N=1,843)*.* ^Remoteness indicators are based on 2016 ABS data, and as such, individuals who reside in newer postcodes established after 2016 (n=2 in analysis sample; n=37 in full sample) are missing data on this variable. ^†^Based on Single Item Literacy Screener (SILS): How confident are you with filling out medical forms by yourself: not at all, a little bit, somewhat, quite a bit, extremely. “Not at all” response categorised as inadequate health literacy.
