# Supplementary material for: Contextualising COVID-19 prevention behaviour over time in Australia: Patterns and long-term predictors from April to July 2020 in an online social media sample
Source: PLoS One. 2021 Jun 29;16(6):e0253930. doi: 10.1371/journal.pone.0253930 (PMC8241082; doi:10.1371/journal.pone.0253930)
Supplement: S2 Table — Values are presented as estimated mean differences from the fixed portion of the linear mixed models, and can be interpreted as standard deviation units. (DOCX) [file pone.0253930.s002.docx]

**S2 Table. Sensitivity analyses of pairwise comparisons between the April (baseline) and subsequent surveys on distancing and hygiene component scores (i.e., ‘stay at home’ behaviour not included in the PCA). Values are presented as estimated mean differences from the fixed portion of the linear mixed models, and can be interpreted as standard deviation units.**

| Pairwise comparisons to April Survey (baseline) | Component 1: Distancing  Estimated mean difference (95% CI); p-value | Component 2: Hygiene  Estimated mean difference (95% CI); p-value |
| --- | --- | --- |
| May Survey | -0.28 (-0.34, -0.23), p<.001 | -0.14 (-0.18, -0.09), p<.001 |
| June Survey | -0.69 (-0.77, -0.61), p<.001 | -0.14 (-0.19, -0.09), p<.001 |
| July Survey | -0.80 (-0.88, -0.72), p<.001 | -0.14 (-0.20, -0.09), p<.001 |
