# Supplementary material for: Contextualising COVID-19 prevention behaviour over time in Australia: Patterns and long-term predictors from April to July 2020 in an online social media sample
Source: PLoS One. 2021 Jun 29;16(6):e0253930. doi: 10.1371/journal.pone.0253930 (PMC8241082; doi:10.1371/journal.pone.0253930)
Supplement: Attachment — Submitted filename: response to reviewer comments.pdf [file pone.0253930.s004.pdf]

Dear Arista Lahiri,

RE: PONE-D-21-07536 Contextualising COVID-19 prevention behaviour over time in Australia: Patterns and long-term predictors from April to July 2020

Thank you very much for the opportunity to revise our article submitted to PLOS ONE. We have read the comments of the reviewers with interest, and have revised our paper in light of these comments. We have addressed each comment, with full details of the revisions given below in bold. These are also shown in track changes in the manuscript document.

Journal Requirements:

When submitting your revision, we need you to address these additional requirements.

1. Please ensure that your manuscript meets PLOS ONE's style requirements, including those for file naming. The PLOS ONE style templates can be found at

https://journals.plos.org/plosone/s/file?id=wjVg/PLOSOne_formatting_sample_main_body.pdf and

https://journals.plos.org/plosone/s/file?id=ba62/PLOSOne_formatting_sample_title_authors_affiliations.pdf

This is now done.

2. We note that you have indicated that data from this study are available upon request. PLOS only allows data to be available upon request if there are legal or ethical restrictions on sharing data publicly. For information on unacceptable data access restrictions, please see http://journals.plos.org/plosone/s/data-availability#loc-unacceptable-data-access-restrictions.

In your revised cover letter, please address the following prompts:

a) If there are ethical or legal restrictions on sharing a de-identified data set, please explain them in detail (e.g., data contain potentially identifying or sensitive patient information) and who has imposed them (e.g., an ethics committee). Please also provide contact information for a data access committee, ethics committee, or other institutional body to which data requests may be sent.

b) If there are no restrictions, please upload the minimal anonymized data set necessary to replicate your study findings as either Supporting Information files or to a stable, public repository and provide us with the relevant URLs, DOIs, or accession numbers. Please see http://www.bmj.com/content/340/bmj.c181.long for guidelines on how to de-identify and prepare clinical data for publication. For a list of acceptable repositories, please see http://journals.plos.org/plosone/s/data-availability#loc-recommended-repositories.

This is now done. Deidentified data will be made available as a supporting file (S2).

We will update your Data Availability statement on your behalf to reflect the information you provide.

This is now done. The Data Availability Statement reads “Supporting data are available from S2.”

3. Please amend either the abstract on the online submission form (via Edit Submission) or the abstract in the manuscript so that they are identical.

This is now done.

4. Please include captions for your Supporting Information files at the end of your manuscript, and update any in-text citations to match accordingly. Please see our Supporting Information guidelines for more information: http://journals.plos.org/plosone/s/supporting-information

This has been corrected.

Additional Editor Comments:

The authors have presented a well-written piece of evidence regarding COVID-preventive behaviors in Australia over the months of April to July, 2020. They have attempted to describe the changing pattern of these behvaiors over time and also sought to understand the role of certain behavioral predictors. However, there are certain concerns and the article needs further clarification on the following issues:

1. Sampling of participants and representativeness of the sample. The authors must also revise their title, because I am afraid the findings do not represent the whole Australian population, rather only a digitally advanced group of people were studied. This part needs clarification and requisite modifications.

Thank you for this comment We have revised the title to better reflect the sample:
"*Contextualising COVID-19 prevention behaviour over time in Australia: Patterns and long-term predictors from April to July 2020* ***in an online social media sample."***

We do acknowledge the limitations of a sample recruited through social media, including in the discussion. Whilst this particular sample has overrepresentation of females and university-educated Australians, we were able to sample people from diverse age ranges, regional areas, and with varying health status, as shown in Table 2 in the manuscript.

2. Considering the study-tool to be a non-standard one, detailed validity and reliability information is needed.

The 7 behaviour items used to derive the outcome measures at baseline had moderate internal consistency (reliability) as reflected by a Cronbach's alpha of 0.72 with appropriate sampling adequacy as reflected by a Kaiser-Meyer-Olkin (KMO) Measure of Sampling Adequacy value of 0.78, suggesting that the data were suitable for principal components analyses (this information is conveyed on pg 13). As a data-driven approach (PCA with rotation) was used to identify and extract the two behavioural components, it is not surprising that individual items were moderately correlated with the component that they loaded greatest on to (see Table 4; p14), and that the correlation was stronger with the component loaded greatest onto compared to the component with weaker loading – reflecting convergent and divergent validity, respectively. Notably, this pattern of correlation was maintained across all survey time points. These correlations are provided below for the interest of the editor and reviewers. Concurrent validity cannot be assessed as there are no similar, validated measures included in the dataset with which to compare; note however, that this approach was used as a way of reducing the dimensionality of the data for analysis, rather than developing a scale.

Table 1. Correlation coefficients between individual behaviour items and extracted behavioural components at each survey time point. Values in bold reflect the component onto which the individual item loaded most strongly onto at baseline (see Table 4 in manuscript for component loadings).

	Distancing Behaviour	Hygiene Behaviour
**Baseline (April Survey)**		
Staying at home	**0.72**	0.13
Washing hands frequently	0.55	**0.64**
staying 1.5m away from other people	**0.74**	0.25
Avoiding close contact with anyone with cold or flu like symptoms	**0.60**	0.36
Avoiding touching face	0.42	**0.79**
Cleaning frequently touched surfaces	0.16	**0.81**
Avoiding shaking hands, hugging or kissing as a greeting	**0.59**	0.37
**May Survey**		
Staying at home	**0.71**	0.13
Washing hands frequently	0.51	**0.69**
staying 1.5m away from other people	**0.72**	0.27
Avoiding close contact with anyone with cold or flu like symptoms	**0.56**	0.34
Avoiding touching face	0.43	**0.79**
Cleaning frequently touched surfaces	0.22	**0.80**
Avoiding shaking hands, hugging or kissing as a greeting	**0.62**	0.38
**June Survey**		
Staying at home	**0.78**	0.15
Washing hands frequently	0.52	**0.69**
staying 1.5m away from other people	**0.77**	0.35
Avoiding close contact with anyone with cold or flu like symptoms	**0.60**	0.45
Avoiding touching face	0.47	**0.80**
Cleaning frequently touched surfaces	0.29	**0.79**
Avoiding shaking hands, hugging or kissing as a greeting	**0.70**	0.44
**July Survey**		
Staying at home	**0.83**	0.18
Washing hands frequently	0.50	**0.68**
staying 1.5m away from other people	**0.76**	0.38
Avoiding close contact with anyone with cold or flu like symptoms	**0.52**	0.43
Avoiding touching face	0.51	**0.81**
Cleaning frequently touched surfaces	0.31	**0.80**
Avoiding shaking hands, hugging or kissing as a greeting	**0.71**	0.41

3. The non-response and drop out in the study appears to be high. Did the authors compare the basic characteristics between the responders and non-responders?

A comparison of the full and analysed sample is provided in Appendix Table 1. We have added text to the Statistical analysis section of the methods to elaborate on this further:

> *The analysis sample comprised participants from our prospective longitudinal study (25) who provided responses to the baseline survey (April) and at least one subsequent follow-up survey (N=1,843). A comparison of the full and analysed sample is provided in **S1** Table 1. **No new participants were added at subsequent time points. Participants in the analysis sample were similar to those of the full sample, though a higher proportion were female (71.7% vs 63.5% female) and older (27.1% aged 56 to 90 years vs 23.3%).***

4. Among the respondents in different months, were they the same respondents as from previous months, or were there any new respondents? Authors should make this part clearer. Because based on this the analytical strategy may change.

All participants in the analysis sample completed baseline and at least one additional survey. No new participants were added at subsequent time points who had not completed the baseline survey. We have clarified this in text on p 9. We now detail this in the Methods section:

> *The analysis sample comprised participants from our prospective longitudinal study (25) who provided responses to the baseline survey (April) and at least one subsequent follow-up survey (N=1,843).* ***No new participants were added at subsequent time points****.*

5. The authors have taken into account several COVID-preventive behaviors and based on the responses they have grouped the behaviors in two major themes i.e., distancing and hygiene. Now, in the regression models, the authors have developed separate models for every month of survey. The variables in the regression analyses I feel, may suffer from endogeneity. Authors may please look into it.

The outcome variables in the regression models (distancing and hygiene behaviours in June and July) are continuous variables, calculated using the component loadings determined from principal components analysis at baseline (April) applied to the behaviours reported (measured as Likert scales) at subsequent study time points. The regression analyses control for baseline (April) behaviours (as a component score) to rule out regression to the mean as a factor driving the observed changes over time – however, the models for distancing only includes the baseline distancing score, and the models for hygiene only include the baseline hygiene scores, so endogeneity due to codetermined variables (simultaneity) is not believed to be an issue. Covariate inclusion has been clarified by providing more detail in the footnote of Table 6:

> * Models adjusted for baseline (April Survey) behaviours (distancing or hygiene), age, gender, socioeconomic status, residential remoteness, education, residential state, and health comorbidities. **Models are adjusted for the corresponding baseline behaviour component (i.e. models for distancing behaviours are adjusted for baseline distancing behaviours but not baseline hygiene behaviours).**

6. In order to understand trend or patterns it might have been better to develop a regression model with time as a predictor. In the current study, a multi-level model can be used to make the findings more robust, crisp, and comprehensive. Though I have reservation regarding use of logistic regression only, as the method of analysis in a study where the authors aimed to see the patterns and the predictors, still the use of other applicable or rather more appropriate models is dependent upon the data collection and sampling itself (refer point 4).

Indeed, we have included an analysis utilising linear mixed modelling with random intercepts by participant, which explores how the behaviours (as continuous scores) changed over time (see p14 and Figure 2), which also allows for missing observations at different study time points across the sample. For the regression models identifying baseline factors associated with distancing and hygiene behaviours in June and July (p15/16), linear models (not logistic regression) were employed. While a model incorporating time as a predictor (rather than separate models for June and July) would be more robust due to the larger number of observations included and minimise the number of models presented overall, this would also require fitting interaction terms for time with all explanatory and control variables, substantially increasing the complexity of the model and its interpretability and potentially leading to overfitting. Although it may be slightly less efficient, we feel that the presented models are interpretable without detracting from the

manuscript's overall study findings that, collectively, distancing behaviours changed over time, whereas hygiene behaviours remain similar over time, and that behaviours in June and July were predicted by variables in April including perceived risk of COVID-19, social obligation, self-efficacy, and belief in the effectiveness of the behaviour in preventing infection.

7. In abstract authors may please follow this format: background, objectives, methods, results, conclusion.
The abstract has been revised:

> ***Background:*** *In Australia in March 2020 a national public health directive required that non-essential workers stay at home, except for essential activities. These restrictions began easing in May 2020 as community transmission slowed.*
>
> ***Objectives:*** *This study investigated changes in COVID prevention behaviours from April-July 2020, and psychosocial predictors of these behaviours.*
>
> ***Methods: An*** *Australia-wide* ***(national) survey was*** *conducted in April, with monthly follow-up over four months.* ***Participants who were*** *adults (18+ years), currently residing in Australia and who could read and understand English* ***were eligible****.* ***Recruitment was via online social media.*** *Analysis sample* ***included*** *those who provided responses to the baseline survey (April) and at least one subsequent follow-up survey (N=1834 out of a possible 3216 who completed the April survey). 71.7% of the sample was female (n=1,322). Principal components analysis (PCA) combined self-reported adherence across seven prevention behaviours. PCA identified two behaviour types: 'distancing' (e.g. staying 1.5m away) and 'hygiene' (e.g. washing hands), explaining 28.3% and 24.2% of variance, respectively. Distancing and hygiene behaviours were analysed individually using multivariable regression models.*
>
> *Results: On average, participants agreed with statements of adherence for all behaviours (means all above 4 out of 7). Distancing behaviours declined each month (p's<.001), whereas hygiene behaviours remained relatively stable. For distancing, stronger perceptions of societal risk, self-efficacy to maintain distancing, and greater perceived social obligation at baseline were associated with adherence in June and July (p's<0.05). For hygiene, the only significant correlate of adherence in June and July was belief that one's actions could prevent infection of family members (p<.001).*
>
> *Conclusion: High adherence to COVID prevention behaviours were reported* ***in this social media sample****; however, distancing behaviours tended to decrease over time. Belief in social responsibility may be an important aspect to consider in encouraging distancing behaviours. These findings have implications for managing a shift from government-imposed restrictions to individual responsibility.*

<u>Reviewer #1:</u>

This study highlights the importance of considering how patterns of COVID prevention behaviours change over time, and suggests that different policy approaches especially risk communication approaches, may be needed for different behavioural categories in different stages of the pandemic. The data has been elaborately analysed and presented. Appropriate statistical analysis been performed supporting the conclusions drawn.

The manuscript is written in a lucid and unambiguous form which is very comprehensible.

As a future research direction, the authors could consider analysis of complexity of performance of the seven precautionary behaviors along with their contextual facilitators and barriers.

Thank you for your positive feedback about the manuscript. To address the comment above, we have added the following to the future directions section of the discussion:

> *Future directions*
> *As we observed different patterns over time for hygiene and distancing behaviours, future research should carefully consider which behaviours are included in analyses of COVID prevention behaviours, and whether these behaviours are analysed collectively or independently. For policy makers, our results suggest that environmental cues and messages may be sufficient to maintain new habits for simple hygiene behaviours, but enforcement may be required for more complex social behaviours like distancing as this appears to be more difficult for individuals to regulate and maintain.* ***Future research could investigate the mechanisms by which environmental and psychological factors facilitate or impede habit formation for COVID-19 prevention behaviours and the effectiveness of enforcement strategies.***

Reviewer #2:

Overall paper is good.
1. Sample size seems low
We can provide you with more detail of how the sample size was determined. Baseline sample size was based on comparable cross-sectional US data collected in March, calculated to achieve a specified level of precision (confidence interval width) in point estimates at 6-month assessment in the longitudinal cohort, while also accounting for potential loss to follow-up.

When the study was designed, the item pertaining to societal risk perception of COVID-19 was selected as the primary outcome to form the basis of the power analysis. To achieve 95% confidence intervals no wider than 0.2 units (±0.1 units) for point estimates of the continuous outcome 'societal risk perception' (1-10 scale) was estimated to require a minimum of 1111 participants. This number of participants would also allow for 95% confidence intervals around point estimates of proportions to have width no greater than ±3%. We assumed loss-to-follow-up of 20% for initial assessment timepoints up to one month (i.e. 80% of the initially recruited sample is retained at 2 weeks, and 80% of these participants are retained at one month), a further 20% loss at 3-months, and 20% at 6-months; leaving approximately 40% of the initially recruited sample by the 6-month assessment.

Therefore, to have the desired number of participants at 6-months required ~2778 participants to be initially recruited at baseline (i.e., an inflation factor of 2.5 of the desired sample size) for this longitudinal cohort. The full sample for this longitudinal cohort, as show in Appendix Table 1, was 3,216. A sample of this size was also estimated to provide 80% power (with alpha = 0.05) to detect small effect sizes (Cohen's d = 0.17) for pairwise comparisons between groups of interest for continuous outcomes, and differences in proportions between groups of interest as small as 8.5%; as well as providing ample power to conduct cross-sectional and longitudinal multivariable regression modelling.

2. Citation format issue on page 15 Betsch, Schmid, Heinemeier, Korn, Holtmann and Böhm (30)
This has been corrected in the manuscript.

3. NSW and the Australian Capital Territory is over represented in collected data otherwise sampling is very good.
The relatively high proportion of NSW residents may reflect that the social media post used the University of Sydney logo. It is possible that this attracted more NSW participants because they were more familiar with the university than people from other states/territories. We have included a comment on this in the limitations:

> *We also acknowledge that recruitment via social media has resulted in a sample that is younger, more educated and has a higher proportion of women than the Australian population. Results may not generalise to older, less educated samples with a higher proportion of men [35].* ***Similarly, although the sample reflected national distributions in remoteness, a larger proportion of the sample resided in NSW (51%) compared to the population (32%). Given that NSW had more cases than other states/territories excluding Victoria, and given that policies differed by state, findings in this study may not apply as strongly to other states with fewer local cases.*** *The regression models presented in the results do control for these factors but may not entirely remove this artifact. This sample consented to take part in a longitudinal study on COVID-19 and so may be more likely to have an active interest in COVID-19. As such, participants in this sample may be more engaged in government recommendations for prevention behaviours. Despite these limitations, the extent of COVID prevention behaviours reported in this paper mirrors the high estimates reported in nationally representative surveys [3-7], and the sample is representative on other factors such as remoteness and number of chronic conditions.*

4. On page 22, figure 2 missing on page but included later
PLOS One submission requires figures are removed and provided in separate files.

5. Only overall behavior over time is shown in graphs, not behavior/perceptions separated by demographics.
Whilst sub-analyses by demographics could provide useful information, we feel that for the size of the sample and the number of combinations of demographics and variables that could be presented, this is beyond scope of the current manuscript.

6. Paper doesn't point out if there were other efforts made to get data from people outside Facebook(email marketing, ads on Youtube, etc) but does point out that sampling methods could affect results.
This is correct, this sample was recruited through social media only (Facebook and Instagram), as outlined in the 'Design' section of the Methods. At the same time as the April recruitment, we also collected data for a cross-sectional survey using online panels; this is reported elsewhere[1]. In November 2020 (i.e. after the data in this manuscript was already collected) we recruited a new cross-sectional, nationally representative sample using online panels for another COVID-19 surveys. This approach overcomes the limitations of this social media sample, but does not provide the benefits of longitudinal data.

Additionally, we acknowledge that few participants in the sample analysed in this manuscript came from culturally or linguistically diverse backgrounds. Another project is currently underway to address this gap in Australian research on COVID-19 behaviours, knowledge and attitudes. We have added this to the future directions section:

> ***Future research on COVID-19 behaviours should also consider diverse groups; currently we are recruiting a survey sample across ten language groups in Greater Western Sydney.***

McCaffery K, Dodd R, Cvejic E, et al.: Health literacy and disparities in COVID-19–related knowledge, attitudes, beliefs and behaviours in Australia. Public Health Research & Practice.

Reviewer #3:
I have 2 main concerns with the work:
1) accuracy of self reporting - i am unsure of how accurate self reporting for these behaviours really is as an assessment tool
We acknowledge that self-reported behaviours may be less accurate than objective measures. However, objective measures of COVID-19 behaviours tend to focus on general movement patterns and would not provide data at the level of detail that this paper investigates (e.g. hand washing, avoiding touching one's face, avoid contact with people who have flu-like symptoms). We now report this limitation in the discussion:

> *We also acknowledge this study's limitations. Interpretation of some of these items may have changed over time. For example, the sense of 'distancing' may have changed in Australia between April and July. In April, during national restrictions, government advice on distancing clearly made reference to strangers as well as family and friends. As social venues such as restaurants have opened up, distancing is more likely to refer to strangers and implicitly excludes close friends or family. If this is the case and the 'meaning' of distancing has become less conservative over time, this current analysis may underestimate the decrease in distancing behaviours over time. For example, people may apply the 1.5 metre rule to strangers in public but not when visiting friends or extended family in other homes, a common setting for COVID-19 clusters in Australia.* ***Additionally, self-reported data may be subject to recall and desirability biases that reduce accuracy. However, self-reported behavioural measures have practical advantages and are widely used in COVID-19 research [3-7,12-20].***

2) sample - considering that Australia has roughly 25.3 million people, a sample size of 1834 does not seem representative of the overall population. Moreover the sample encompasses a majority of highly educated women, which again reinforces that the sample is very selective compared to the national australian estimates.
The article is well written but i question the significance of the results based on such a small and non-representative sample and I because of that I don't think these results can be extrapolated to the australian population at large.

We acknowledge that the lack of representativeness of the sample is a limitation of the study, and this is discussed at length in the discussion:

> *We also acknowledge that recruitment via social media has resulted in a sample that is younger, more educated and has a higher proportion of women than the Australian population. Results may not generalise to older, less educated samples with a higher proportion of men.*

Where possible, we have tried to be transparent about the sample not being representative, for example, by comparing sample demographics to those of the Australian population, and by comparing our findings about COVID-19 prevention behaviours to those from surveys implemented by the Australian Bureau of Statistics (ABS). As outlined in the introduction, this paper builds on the findings of the ABS surveys by providing longitudinal data that follows the same seven behaviours over time.

We do not intend to claim that these findings are generalisable to all Australians. To make this clearer, where possible we have revised the language in the title, abstract, discussion and conclusion:

Title*: "Contextualising COVID-19 prevention behaviour over time in Australia: Patterns and long-term predictors from April to July 2020* ***in an online social media sample****"*

Discussion

This study found that in an Australian ***social media sample****, there were consistently high levels of seven self-reported COVID prevention behaviours across a four-month period (April to July, 2020). The observed patterns suggest these behaviours can be characterised as either 'distancing' behaviours (staying at home, staying 1.5m away from others, avoiding people with flu-like symptoms, and avoiding shaking hands, hugging or kissing) or 'hygiene' behaviours (washing hands, avoiding touching your face, and cleaning frequently touched surfaces). These two categories of behaviour were moderately correlated but displayed different trajectories over time. Whilst the extent of hygiene behaviours was similar across time-points, distancing behaviours decreased over time, with the largest decrease observed in July (equivalent to 1.5 standard deviations below the mean level reported for these behaviours in April)…*

Conclusion

In this Australian ***online social media sample****, participants reported consistently high levels of seven COVID prevention behaviours between April and July, 2020. Distancing behaviours decreased over time, whereas hygiene behaviours remained relatively stable. Along with self-efficacy, factors relating to others and the community at large were more likely to predict behaviours than personal considerations. This study highlights the importance of considering how patterns of COVID prevention behaviours change over time, and suggests that different policy approaches may be needed for different behavioural categories.*
